# Specific egg yolk antibody raised to biofilm associated protein (Bap) is protective against murine pneumonia caused by *Acinetobacter baumannii*

**DOI:** 10.1038/s41598-022-16894-w

**Published:** 2022-07-22

**Authors:** Azam Ranjbar, Iraj Rasooli, Abolfazl Jahangiri, Fatemeh Ramezanalizadeh

**Affiliations:** 1 Department of Biology, Shahed University, Tehran-Qom Express Way, Tehran, 3319118651 Iran; 2 Molecular Microbiology Research Center and Department of Biology, Shahed University, Tehran, Iran; 3 Applied Microbiology Research Center, Systems Biology and Poisonings Institute, Baqiyatallah University of Medical Sciences, Tehran, Iran

**Keywords:** Immunology, Microbiology, Infectious diseases

## Abstract

*Acinetobacter baumannii* easily turns into pan drug-resistant (PDR) with a high mortality rate. No effective commercial antibiotic or approved vaccine is available against drug-resistant strains of this pathogen. Egg yolk immunoglobulin (IgY) could be used as a simple and low-cost biotherapeutic against its infections. This study evaluates the prophylactic potential of IgY against *A. baumannii* in a murine pneumonia model. White Leghorn hens were immunized with intramuscular injection of the recombinant biofilm-associated protein (Bap) from *A. baumannii* on days 0, 21, 42, and 63. The reactivity and antibiofilm activity of specific IgYs raised against the Bap was evaluated by indirect ELISA and a microtiter plate assay for biofilm formation. The IgYs against Bap were able to decrease the biofilm formation ability of *A. baumannii* and protect the mice against the challenge of *A. baumannii*. IgYs antibody raised here shows a good antigen-specificity and protectivity which can be used in passive immunotherapy against *A. baumannii*. In conclusion, the IgY against biofilm-associated protein proves prophylactic in a murine pneumonia model.

## Introduction

*Acinetobacter baumannii* is the prime pathogen that urgently needs new antibiotics among the most common nosocomial infectious pathogens known as ESKAPE (*Enterococcus Faecium, Staphylococcus aureus, Klebsiella pneumoniae, A. baumannii, Pseudomonas aeruginosa*, and *Enterobacter* species)^1,2^. This Gram-negative bacterium causes several infection types including urinary tract infections, bacteremia, sepsis, meningitis, endocarditis, and skin and soft tissue infections, especially in patients with acute conditions in the intensive care unit^3^. Long-term hospitalization, old age, weakened immune system, wounds, burns, improper use of antibiotics, and long-term use of catheters and mechanical ventilators are factors that lead to *A. baumannii* infections^4^. The rapid emergence of antibiotic-resistant strains along with a high mortality rate is alarming. The ability to form biofilms helps *A. baumannii* to increase its resistance to antimicrobial agents and environmental stresses^5^. Biofilm formation is a complex process in which many protein virulence factors are involved such as pilli, outer membrane proteins (OMPs), OmpA, Csu pili, Ata Bap, etc^6–9^. The *bap* had been detected in antibiotic-resistant strains of *A. baumannii* with high prevalence^10^. This gene encodes Bap which plays an important role in biofilm formation^11^. This protein is also involved in the adherence of *A. baumannii* to human epithelial cells^12^. Bap with 8620 amino acids and isoelectric point (pI) ~ 3 is one of the largest acidic proteins originally identified in AB307-0294 strain by Loehfelm et al.^13^.

In *A. baumannii*, Bap is secreted by the type I secretory system^14^. This protein harbors consecutive repetitive sequences^13^. Four conserved regions of Bap were identified as possessing antigenic properties that could render the identified protein products as appropriate vaccine candidates^14^. Immunization of the conserved region of Bap (706–1061) from *A. baumannii* strain Kh0060 was later evaluated in a murine model by Fattahian et al.^15^. The immunogenic region conferred full protection against 100 × LD_50_ and 80–60% protection against 10^3^–10^5^ × LD_50_ of *A. baumannii* strain Kh0060 in a murine sepsis model^15^. This recombinant subunit of Bap then served as an antigen to produce a specific recombinant nanobody against *A. baumannii*. This nanobody recognized *A. baumannii* ATCC 19606 as well as 60 clinical isolates of *A. baumannii*^16^.

The mice were immunized intranasally with chitosan particles harboring the recombinant subunit of Bap (706–1061 region). Passive immunization with sera obtained from these mice conferred 100% protection against 10 × LD_50_ of *A. baumannii* ATCC 19606 in a neutropenic murine pneumonia model.

De Gregorio et al.^17^ analyzed Bap in 541 *A. baumannii* sequenced strains. Their study indicated that Bap was highly polymorphic and was absent among 20% of the sequenced strains. Although Bap is not prevalent in all strains and isolates of *A. baumannii*^17,18^, it is an antigen of interest to be involved in multi-subunit/multivalent antigens. Hence, it is imperative to introduce novel effective solutions against *A. baumannii* infections. Recently, specific egg yolk antibodies (IgYs) had been nominated as promising biological macromolecules to be used against *A. baumannii* pneumonia infection^19,20^. Specific anti-*P. aeruginosa* IgY successfully advanced to a phase III clinical trial in cystic fibrosis patients (ClinicalTrials.gov Identifier: NCT01455675)^21^. The use of IgYs provides several advantages in comparison to its mammalian counterpart, IgG. Production of IgYs needs no invasive and stressful bleeding of the animal. Egg yolk contains a high amount of IgY which could be purified by simple, cost-effective, and environmental-friendly methods^22,23^. It is a safe antibody for passive immunization since it has no interaction with complement and mammalian Fc receptors^23–25^. Although it had been revealed that specific IgYs develop protection against *A. baumannii*, the protection level depends on the antigen of interest^20^. The maximum protection would be expected to be achieved by the combination of the most protective antigens. Hence, further studies to evaluate various antigens are warranted. In the current study, the protectivity of specific anti-Bap IgYs against *A. baumannii* is assessed in a murine pneumonia model. In this regard, a 371-amino acid region of Bap was recombinantly overexpressed, purified, and injected into laying hens. A clinical isolate of *A. baumannii* was used to develop a murine pneumonia model. Prophylactic effects of obtained specific antibodies were studied in the developed murine pneumonia model.

## Results

### Prepared antigen and IgY antibodies

**Figure 1:**
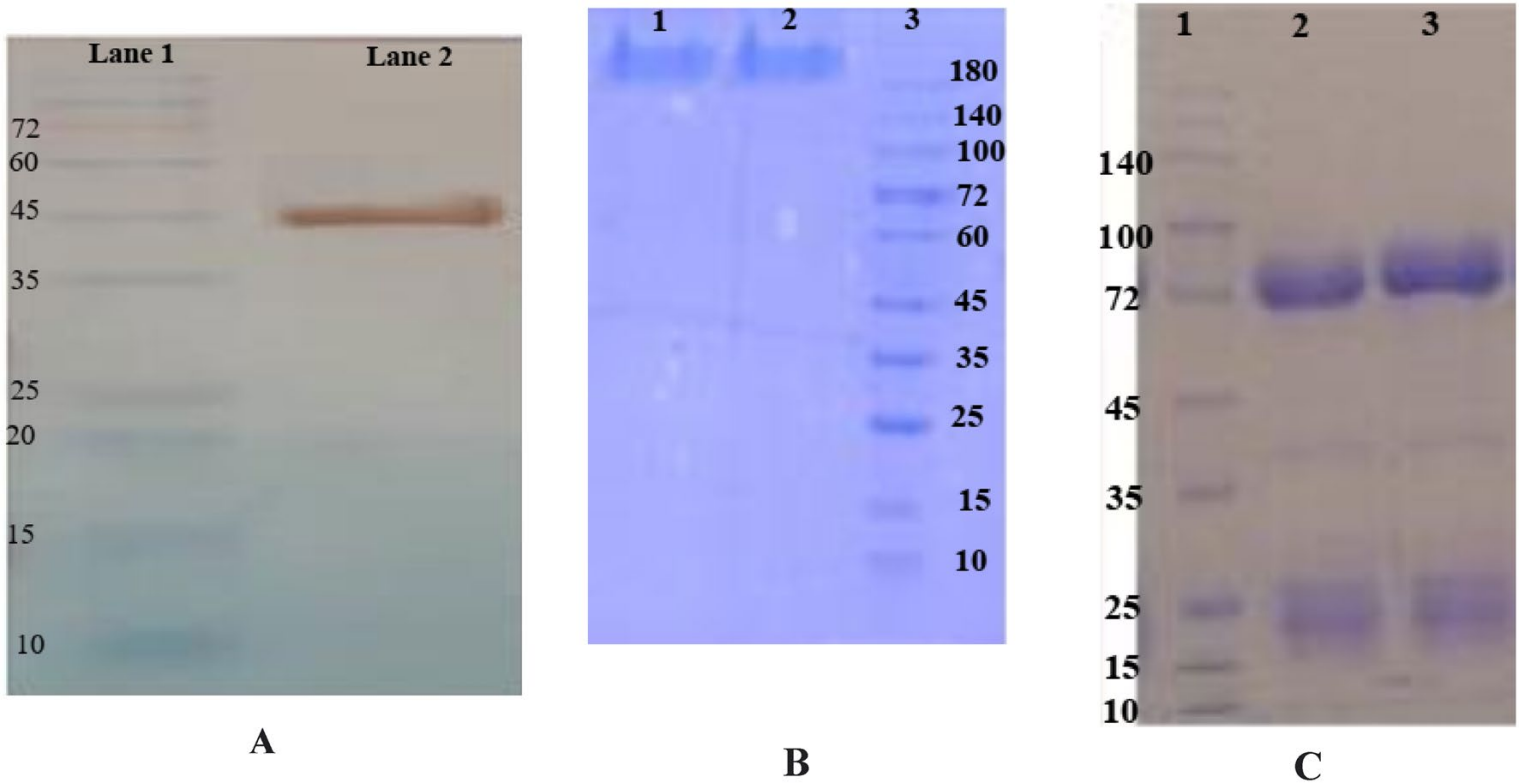
The confirmation of recombinant protein (Bap) expression by western blotting analysis (**A**), and purified IgY antibodies by SDS-PAGE analysis (**B**, **C**). (**A**) The result showed a band with a molecular weight of 45 kDa, Lane 1: Protein marker, Lane 2: Bap. (**B**) The confirmation of purified IgY antibodies by SDS-PAGE analysis under the non-reducing condition, 10% SDS-PAGE gel showed a band (180 kDa), Lane 1: IgY-Bap, Lane 2: IgY- control, Lane 3: Protein marker. (**C**) The confirmation of purified IgY antibodies by SDS-PAGE analysis under the reducing condition, two bands on 10% SDS-PAGE gel are the heavy chain (75 kDa) and the light chain (25 kDa) of IgY, Lane 1: Protein marker, Lane 2: IgY-Bap, Lane 3: IgY- Control.

SDS-PAGE analysis showed high purity of the recombinant subunit Bap with a molecular weight of 45 kDa. Western blotting by Anti-His HRP-conjugated antibodies confirmed the recombinant Bap protein (Fig. 1A). SDS-PAGE analysis of purified IgY antibodies revealed their yield and purity (Fig. 1B, C).

### Immunoassay results

**Figure 2:**
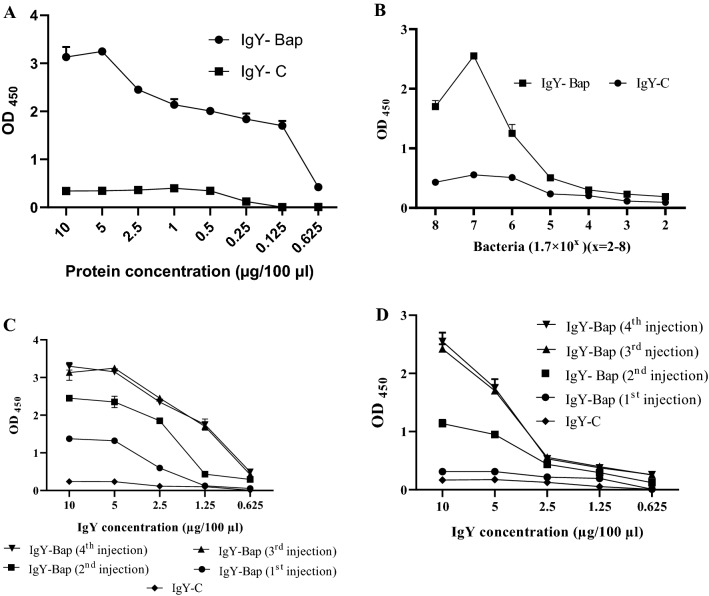
Indirect ELISA. (**A**) Effect of protein concentration in indirect ELISA against 10 µg IgY. (**B**) Effect of bacterial load in whole-cell ELISA against 10 µg IgY. The horizontal axis shows the number of bacteria ranging from 1.7 × 10^2^ to 1.7 × 10^8^. (**C**) Indirect ELISA of IgY against 5 µg of Bap coated in each well. (**D**) Indirect ELISA of IgY against 1.7 × 10^7^ CFU whole-cells of *A. baumannii* Kh0060 coated in each well. The values represent mean triplicate independent experiments + /− standard error (SEM).

The immunoreactivity of purified IgY raised against the recombinant protein (Bap) is shown in Fig. 2. The results indicated a significant increasing trend of IgY antibody titer against Bap in immunized hens compared to IgY antibody titer in unimmunized hens (*p* < 0.0001) (Fig. 2A, C). Whole-cell ELISA using a clinical strain of *A. baumannii* as an antigen also confirmed the finding of protein ELISA (*p* < 0.0001) (Fig. 2B, D).

### Biofilm inhibition assay

**Figure 3:**
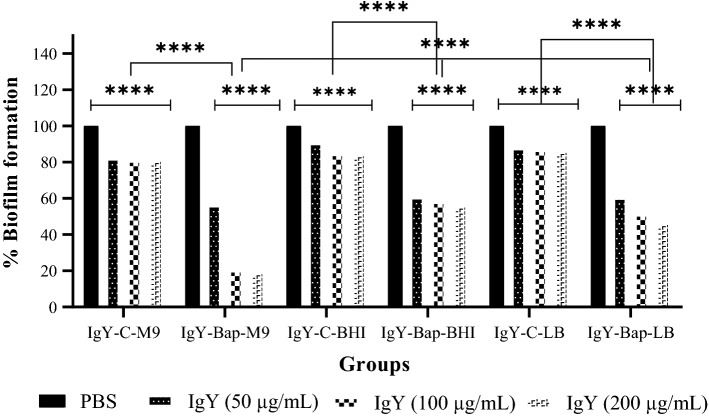
Inhibition of biofilm formation by a clinical strain of *A. baumannii* in M9, BHI, and LB media in the presence of various concentrations of prophylactic IgY. Formation of biofilm in different media against each IgY-Bap group compared with that of PBS as well as IgY-C groups. The IgY-Bap groups were also compared independently and with each other (*p* < 0.0001).

The inhibition of biofilm formation by the clinical strain of *A. baumannii* in 96 wells microplates in the presence of IgY antibodies in LB, BHI, and M9 media is shown in Fig. 3. According to the results, the percentage of biofilm formation in IgY-Bap groups decreased significantly compared to the PBS and IgY-C groups. In the IgY-Bap groups increasing of IgY-Bap antibody concentration, significantly decreased biofilm formation, particularly in the M9 medium.

### Biofilm disruption assay

**Figure 4:**
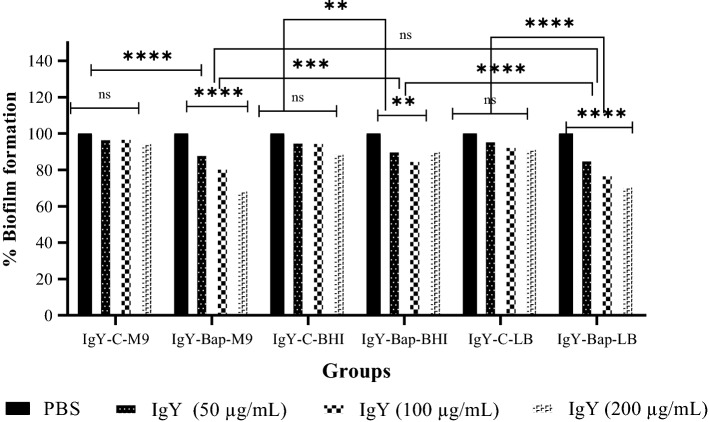
Effect of therapeutic IgY on biofilm disruption in M9, BHI, and LB media. Formation of biofilm in different media against each IgY-Bap group was compared with that of PBS as well as IgY-C groups. The IgY-Bap groups were also compared separately as well as with each other (ns; not significant, **p < 0.01; ***p < 0.001; ****p < 0.0001).

The percentage of disruption of biofilm formed in 96 wells microplates by a clinical strain of *A. baumannii* was evaluated in the presence of IgY antibodies in LB, BHI, and M9 media. The percentage of biofilm disruption in IgY-Bap groups increased significantly compared to the PBS and IgY-C groups (Fig. 4). IgY-Bap-BHI group showed the least amount of disruption.

### Challenge in a murine pneumonia model

**Figure 5:**
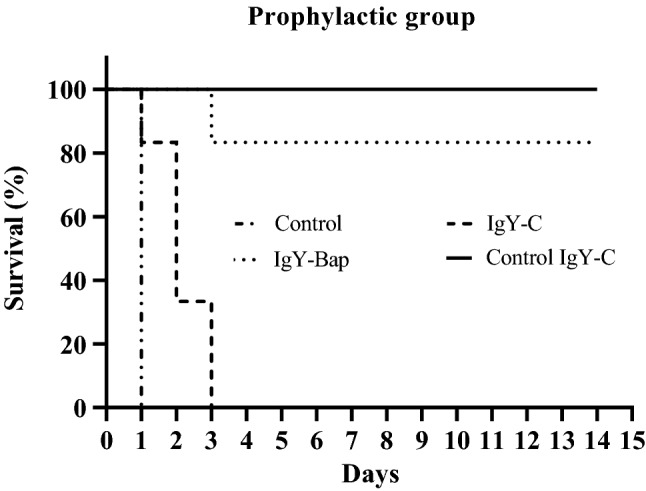
Mice survival rate followed up for 14 days after the challenge in the prophylactic mode. Control: The control group received only *A. baumannii* (Kh0060), IgY-C: Mice received control IgY (IgY-C) 1 h before inoculation with *A. baumannii*, IgY-Bap: Mice received IgY-Bap antibody before inoculation with *A. baumannii*, and Control IgY-C: Mice received only IgY-C antibody (no bacterial challenge).

**Figure 6:**
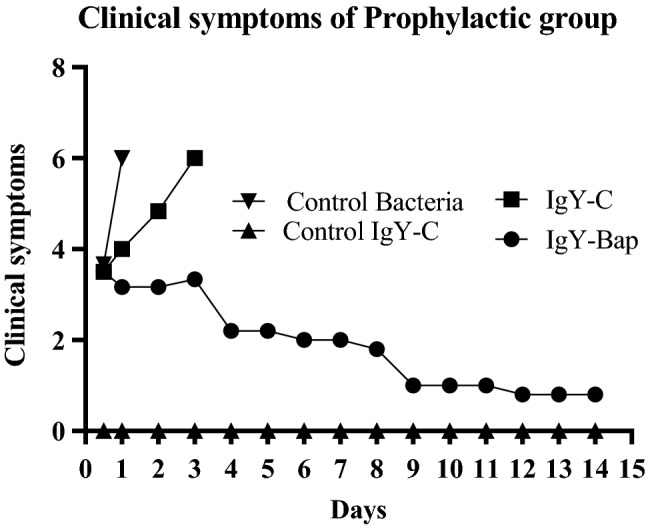
Clinical symptoms of mice for 14 days after challenge. Control: The group without any treatment before infection. IgY-C: the group receiving control IgY 1 h before inoculation. IgY-Bap: the group receiving IgY-Bap antibody 1 h before inoculation. Control Igy-C: the group receiving only the control IgY-C antibody. No clinical sign was seen in the control IgY-C group. In the IgY-Bap group, the mean clinical symptoms after 72 h showed a descending trend compared to the IgY-C and bacterial groups. The recovery process began with the reduction of the severity of symptoms.

**Figure 7:**
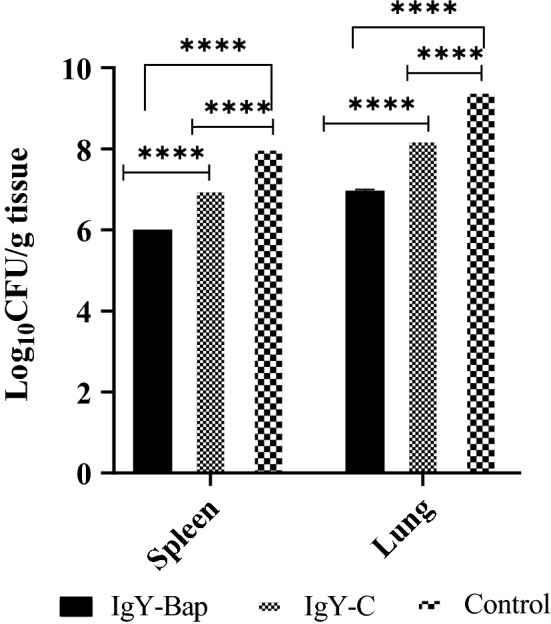
Bacterial load per gram spleen or lung tissue 16 h after challenge. The bacterial load was 2 log_10_ CFU/g less than the control group, and 1 log_10_ CFU/g less than the IgY-C group after 16 h. A significant difference (*p* < 0.0001) was observed between the IgY-Bap group and control bacteria group as well as the IgY-C group 16 h after the challenge. Such difference was also observed between the IgY-C group and the bacteria group (*p* < 0.0001). Control: The group without any treatment before infection. IgY-C: the group receiving control IgY antibody 1 h before inoculation with live bacterial suspension. IgY-Bap: the group receiving IgY-Bap antibody 1 h before inoculation. IgY-C: the group receiving only control IgY-C antibody 1 h before inoculation. Control Bacteria group: The group without any treatment before inoculation (****p < 0.0001).

LD_50_ was determined as 1.7 × 10^7^ CFU. The control IgY-C group receiving only IgY-C antibody (no bacterial challenge), survived for up to 14 days of monitoring. 83% survival was noted in the IgY-Bap group administered with IgY-Bap antibody. All control mice receiving only bacteria died within the first 24 h. All mice receiving IgY-C before the challenge died within 72 h (Fig. 5). Clinical symptoms observed during the 14 days of monitoring included a scruffy coat, squinted eyes, weakness, reduced activity, hunched posture, limping, and finally death (Fig. 6). The mean grade of clinical signs was assessed^20^. Sixteen hours after the inoculation of bacteria, the number of bacteria in the lung and spleen of mice of the IgY-Bap group was at least 2 log_10_ CFU/g less than the control group that received only PBS before the challenge (Fig. 7, and Table 1).

**Table 1:** The number of bacteria per gram of spleen and lung tissue 16 h after challenge in each mice group.

Groups	Bacterial count (CFU/g) in tissues	
	Lung	Spleen
Control bacteria	3.03 × 10^9^	1.5 × 10^8^
IgY-C	2.03 × 10^8^	1.02 × 10^7^
IgY-bap	1.05 × 10^7^	1.02 × 10^6^

## Discussion

*Acinetobacter baumannii* is a gram-negative coccobacillus considered one of the most notorious nosocomial pathogens. Due to the emergence of antibiotic-resistant strains of *A. baumannii*, the use of non-antibiotic methods such as active and inactive immunization by a vaccine is appreciated. Hence, immunotherapy methods such as the use of polyclonal antibodies (IgY) could be promising against *A. baumannii* infections. Active immunization of mice with a region of Bap (706–1061 region) could lead to protection in the mice sepsis model against *A. baumannii* Kh0060^15^. Recently, passive immunization using IgYs raised against the whole cell, OmpA and Omp34 were assessed and showed protection in the pneumonia model. The finding showed an increased survival rate with IgYs against the recombinant protein in comparison to the whole-cell immunization^15,19,20^. Whole-cell ELISA demonstrated that specific IgY antibodies raised against the recombinant subunit Bap could recognize the Bap exposed on the cell surface of *A. baumannii*. However, as expected, the absorbance of whole-cell ELISA was lower than the purified protein. These findings are consistent with the previous investigations^15,19,20^. The selected region is covering less than 5% of the Bap sequence. Expression level and the number of Bap expressed on *A. baumannii* are also involved in the observed absorbance of whole-cell ELISA. ELISA results showed no significant difference between titers of specific antibodies raised after the third and fourth injections. Three injections with 3-week intervals are sufficient to reach the highest titer of anti-Bap IgYs. Specific anti-Bap IgY could decrease biofilm formation in M9, LB, and BHI media. Although 50 μg/mL of the specific IgY showed about 40–50% decrease in biofilm formation in M9, LB, and BHI media, 100 and 200 μg/mL of the specific IgY in various media showed significant variations (> 40–80%). The most inhibition of biofilm formation was observed in the M9 medium; however, the difference between 100 and 200 μg/mL of the specific IgY was not significant. The different anti-biofilm activities of specific anti-Bap IgY in different media could be attributed to different expression patterns of *A. baumannii* proteins including Bap in various conditions^26,27^. Inhibition of biofilm formation by antibodies had been reported previously^15^. Notably, the biofilm-disrupting effects of specific anti-Bap IgY were lower than its effect on inhibition of biofilm formation. So, it could be deduced that prophylactic administration of specific anti-Bap IgY is more protective than its therapeutic application. It had been demonstrated a human monoclonal antibody with a biofilm-disrupting effect could enhance antibiotic efficacy against *A. baumannii* infection^28^.

In a recent study, mice intranasally immunized with Bap-loaded chitosan particles revealed higher titers of anti-Bap IgG and IgA antibodies in sera as well as lung and fecal samples in comparison to mice immunized with Bap^29^. Titers of IgA in mice subcutaneously immunized with Bap had not been detectable. Passive immunization with sera obtained from mice subcutaneously received Bap had conferred 67% and 34% protection against 5 × LD_50_ and 10 × LD_50_ respectively. Notably, sera obtained from mice intranasally immunized with Bap-chitosan particles had conferred 100% protection^29^. It seems that the specific anti-Bap IgA could enhance the developed protection against *A. baumannii*. So, different isotypes of antibodies could confer various protections. The survival rate (83%) through passive immunization by anti-Bap specific IgY in our study, shows higher protectivity in comparison to protectivity conferred by anti-Bap specific IgG^29^. It could be attributed to different strains of *A. baumannii* using the number of specific antibodies, and/or intrinsic nature and potency of IgY. Further studies need to be conducted to address this issue. Our previous study showed that 20 μg of specific anti-*A. baumannii* IgYs administered one hour before nasal challenge with viable bacteria could not protect the mice against 10 × LD_50_ of *A. baumannii* ATCC 19606 while 40 μg of these specific IgYs protected the pneumonic mice^19^. No significant difference was reported in the protection conferred by 40 μg and 100 μg of specific anti-*A. baumannii* IgYs administered 4 h post-challenge with 10 × LD_50_ of *A. baumannii* ATCC 19606^20^. However, the protectivity of higher doses of specific IgYs administered before challenge with viable bacteria remains to be explored. Nasal administration volume is a limitation for the evaluation of high doses of IgY. IgY-C could limit the accumulation of bacteria in organs significantly as observed in the first 16 h. Decreased bacterial load resulted in delayed death in this group for 72 h. The death of IgY-C group in prophylactic experiments suggests an increasing trend of bacterial population leading to the death of animals in this group after 72 h of challenge. The survival of IgY-Bap group while lungs and liver were loaded with a lower burden of live bacteria in the first 16 h ultimately leading to 83% survival indicates the efficacy of the administered antibodies in the clearance of bacteria from the lungs and liver. This is further supported by the observations of the clinical symptoms where IgY-C group tends to exhibit worsening heath and the IgY-Bap group tends to recover from the infection. Further studies are needed to assess the immune response and decreasing mortality rate by monitoring the bacterial burden in both groups for at least 72 h. The present results, although with limitations, provide evidence for the favorable effect of IgY as a prophylactic and therapeutic modality against *A. baumannii*. Yet, more research pieces of evidence with animal studies aimed to utilize IgY are vital. Neutrophils are one of the most important factors in the mechanism of action of IgY^30–32^. Since we used neutropenic mice in our study, this mechanism is undermined and the protective effect of IgY could be attributed to the inhibition of biofilm formation and bacterial attachment to the host cell. It would be expected that the protective effect of IgY-Bap be more pronounced in healthy (non-neutropenic) mice. To the best of our knowledge, this is the first report on the effect of anti-Bap IgY against *A. baumannii* infections. The current study could pave the way for the future studies on passive immunization by specific IgYs against the successful nosocomial pathogen, i.e. *A. baumannii*. In the future studies, various strains of *A. baumannii*, higher doses of IgY as well as the protective effect of IgY-Bap in non-neutropenic murine pneumonia model could be addressed as limitations of the current study.

The results show that specific anti-Bap IgY could prevent biofilm formation of *A. baumannii*. Moreover, passive immunization with this IgY could decrease mortality of pneumonic infection caused by *A. baumannii* in prophylactic mode. Therefore, IgY could be considered promising therapeutics to be entered into clinical phases. Further studies and trials on human subjects could open new perspectives in the application IgY as a therapeutic agent.

## Methods

### Preparation of Bap subunit

*Escherichia coli* BL21 (DE3) harboring 706–1076 region of Bap, cloned into pET28a plasmid^15^ were grown in LB medium (containing 70 μg/ml kanamycin). Protein expression was performed by the autoinduction method as previously described^33^. The expressed recombinant Bap was purified by using Ni–NTA affinity chromatography (Qiagen) in denaturing conditions and then, was dialyzed against PBS to remove urea. The purity of the obtained recombinant protein was evaluated by Sodium Dodecyl Sulfate Polyacrylamide Gel Electrophoresis (SDS-PAGE). The recombinant Bap was confirmed through western blotting with an anti-His tag antibody.

### Immunization of hens

The 25-week-old White Leghorn hens were immunized intramuscularly in four sites of the breast muscle on days 0, 21, 42, and 63. In the first injection, 100 μg of the subunit Bap was mixed with complete Freund’s adjuvant (1:1 v/v) in a total volume of 1000 µl. Subsequent boosters were performed with 100 μg of the Bap protein in incomplete Freund’s adjuvant (1:1 v/v). For the control group, a mixture of PBS and Freund adjuvant (complete or incomplete) in a total volume of 1000 μl was administered. Two weeks after each injection, the eggs were collected daily and stored at 4 °C^19^.

### Preparation and specificity of IgY antibody

The separated egg yolk was diluted (1:7 ratio) with distilled water. PH of the diluted egg yolk was adjusted to 5 with 0.5 M HCl. The mixture was frozen at − 20 °C for 72 h and then was filtered on Whatman cellulose filter paper (Sigma-Aldrich) at room temperature (RT) to remove egg yolk fat. NaCl (8.8%) was added to the clear filtrate and its pH was adjusted to 4. The mixture was stirred for 2 h at RT and then centrifuged at 3700 g for 20 min at 4 °C. The pellet was dissolved in phosphate-buffered saline (PBS, pH 7.4)^34^. The purity and size of IgY were monitored by 10% (w/v) SDS-PAGE. The total concentration of the purified IgY was measured using the Bradford method.

### Assessment of specific IgY antibody titers

Reactivity of specific IgY against whole-cell *A. baumannii* or the recombinant Bap subunit was assessed by indirect ELISA. Firstly, effect of antigen concentrations (Bap or whole bacterial cell) was evaluated against 10 µg of IgY. In this regard 1.7 × 10^2^–1.7 × 10^8^ CFU of a clinical strain of *A. baumannii* (Kh0060)^15^ or 0.0625–10 µg of Bap were coated in a 96 well microplate and incubated overnight at 4 °C. The plate was washed × 3 with 0.5% Tween-PBS (PBST) and was then blocked with 5% skim milk solution in PBST. IgY antibodies at 100 µg/mL concentrations were added to wells. The plate was washed × 3 with PBST after the incubation period was over and was then incubated for one h at 37 °C with 100 μL of 1:1000-diluted HRP-conjugated anti-IgY antibody. The Plate was then washed × 3 with PBST, and TMB substrate (Sigma-Aldrich) was added (100 μL/well; at RT) until the negative control group got colored. Color development was stopped with 2 M H_2_SO_4_, and the absorbance was read at 450 nm. For the main indirect ELISAs, 1.7 × 10^7^ CFU of *A. baumannii* (Kh0060)^15^ or 5 µg of Bap were coated in a 96 well microplate and incubated overnight at 4 °C. The remaining steps were carried out as described above except for the concentrations of IgY antibodies which were 6.25, 12.5, 25.0, 50.0, and 100 µg/mL.

### Biofilm inhibition assay

Overnight culture of *A. baumannii* the clinical strain (Kh0060) was inoculated in fresh LB broth medium and allowed to reach OD_600_ of 0.6. The bacterial suspension was centrifuged and then pelleted. The precipitate was adjusted to a concentration of 0.5 McFarland standard with PBS and then was added to each well of a 96-well polystyrene microtiter plate with a total volume of 50 µl. BHI, LB, or M9 medium containing 1% glucose and various concentrations (50, 100, and 200 µg/mL) of IgYs were added to each well of the 96-well polystyrene microtiter plate in a total volume of 150 µl. The plates were covered and aerobically incubated at 37 °C for 72 h. Next, the plates were washed twice with PBS (pH 7.4) and stained with 100 μl of 0.2% crystal violet (Sigma-Aldrich) for 5 min at RT. The biofilm formation was quantified by measuring the corresponding OD_560_ of the supernatant following solubilization with acetone.

### Biofilm disruption assay

All steps and desired values were per the previous section (Biofilm inhibition assay) except for the IgYs added after 72 h and the plate was incubated at 37 °C for 2 h. The biofilm formation was quantified by measuring the corresponding OD_560_ of the supernatant following solubilization with acetone.

### Mice challenge

In mice challenge, four groups (6 mice/group) of female BALB/c mice (22–25 g) were neutropenized by intraperitoneal injection of cyclophosphamide (150 µg per g animal weight) on the first and second days. On the fourth day, all mice were anesthetized by intraperitoneal injection of ketamine (100 µg kg^−1^) and xylazine (20 µg kg^−1^). One hour before the challenge, all groups were intranasally administered with 40 µg IgYs (anti-Bap IgY: IgY-Bap, or control IgY purified from the unimmune hen: IgY-C) or PBS in a total volume of 20 µl. Then, a 10 × LD_50_ dose of *A. baumannii* (Kh0060) 1.7 × 10^8^ CFU was administered intranasally in the final volume of 20 µl. One group received only IgY-C antibody purified from the unimmunized hen (Control IgY-C). All groups were monitored for clinical signs for up to 14 days.

### Statistical analysis

Statistical analyses were performed by GraphPad Prism 9 (GraphPad Prism version 9.00 for Windows, GraphPad Software, LaJolla, CA, USA). Data were analyzed by one-way analysis of variance with Tukey’s multiple comparison test. Survival data for different mice groups were analyzed using the Mantel–Cox log-rank test. Results were expressed as the Mean ± Standard Deviation (SD), and a *p* value of < 0.05 was considered statistically significant**.**

### Ethics approval

We confirm that this study is reported in accordance with ARRIVE guidelines. The principles stated in the Guide for the Care and Use of Laboratory Animals were followed. The animal care protocol was approved by the ethics committee of Shahed University.

## Data Availability

The datasets generated and/or analyzed during the current study are available from the corresponding author on reasonable request.
